# Unifying proteomic technologies with ProteinProjector

**DOI:** 10.1093/bioadv/vbaf266

**Published:** 2025-10-22

**Authors:** Leah V Schaffer, Mayank Jain, Rami Nasser, Roded Sharan, Trey Ideker

**Affiliations:** Department of Medicine, University of California San Diego, La Jolla, CA 92093, United States; Department of Medicine, University of California San Diego, La Jolla, CA 92093, United States; Blavatnik School of Computer Science and AI, Tel Aviv University, Tel Aviv 69978, Israel; Blavatnik School of Computer Science and AI, Tel Aviv University, Tel Aviv 69978, Israel; Department of Medicine, University of California San Diego, La Jolla, CA 92093, United States; Department of Computer Science and Engineering, University of California San Diego, La Jolla, CA 92093, United States; Department of Bioengineering, University of California San Diego, La Jolla, CA 92093, United States

## Abstract

**Summary:**

Proteomics has developed many approaches to inform the subcellular organization of proteins, each with differing coverage and sensitivity to distinct scales. Here, we develop a self-supervised deep learning framework, ProteinProjector, that flexibly integrates all available data for a protein from any number of modalities, resulting in a unified map of protein position. As initial proof-of-concept we integrate four proteome-wide characterizations of HEK293 human embryonic kidney cells, including protein affinity purification, proximity ligation, and size-exclusion-chromatography mass spectrometry (AP-MS, PL-MS, SEC-MS), as well as protein fluorescent imaging. Map coverage and accuracy grow substantially as new data modes are added, with maximal recovery of known complexes observed when using all four proteomic datasets. We find that ProteinProjector outperforms individual modalities and other integration methods in recovery of orthogonal functional and physical associations not used during training. ProteinProjector provides a foundation for integration of diverse modalities that characterize subcellular structure.

**Availability and implementation:**

ProteinProjector is available as part of the Cell Mapping Toolkit at https://github.com/idekerlab/cellmaps_coembedding.

## 1 Introduction

**Figure 1.**
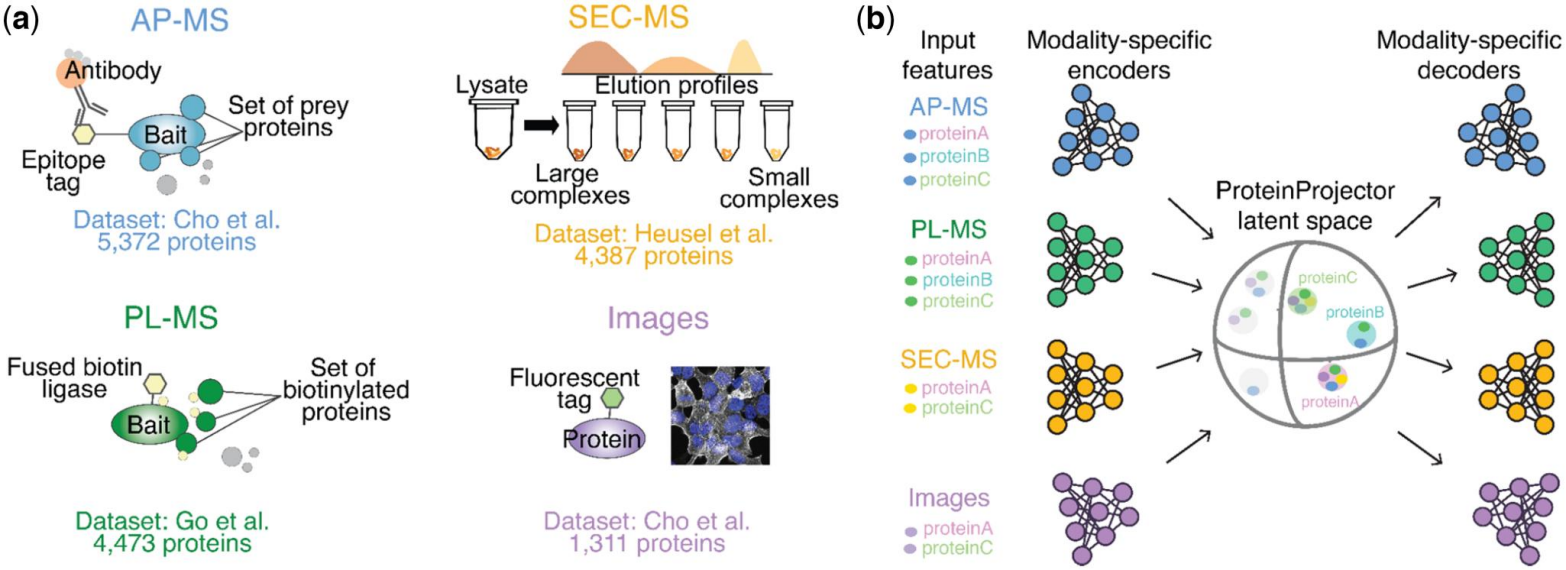
ProteinProjector overview. a) Overview of data modalities obtained from application of proteomic technologies to HEK293 cells: affinity purification (AP-MS), proximity labeling (PL-MS), size exclusion chromatography (SEC-MS), and fluorescence imaging. b) Schematic diagram of ProteinProjector encoder-decoder neural network architecture designed for multi-modal data integration and interpolation.

The last few decades have witnessed enormous advances in proteomic technologies for charting the protein assemblies of human cells (Fig. 1a) (Wilhelm *et al.* 2014, Mulvey *et al.* 2017, Thul and Lindskog 2018, Luck *et al.* 2020, Richards *et al.* 2021, Skinnider *et al.* 2021). For example, affinity purification mass spectrometry (AP-MS) isolates a tagged protein of interest from whole-protein extracts, allowing for the identification of neighboring proteins with biophysical interactions (Huttlin *et al.* 2015, 2021, Gordon *et al.* 2020); proximity labeling mass spectrometry (PL-MS) employs an enzyme fused to a protein of interest to label nearby proteins covalently (Kim *et al.* 2014, Go *et al.* 2021); and size exclusion chromatography mass spectrometry (SEC-MS) identifies groups of proteins with similar elution profiles during chromatography (Havugimana *et al.* 2012, Bludau *et al.* 2020, Fossati *et al.* 2023). Adding to these MS-based approaches, protein fluorescence coupled to confocal microscopy reveals the spatial distribution of a target protein within the cell as well as other proteins that share this distribution (Thul *et al.* 2017, Cho *et al.* 2022). These and numerous other techniques (Geladaki *et al.* 2019, Luck *et al.* 2020, Johnson *et al.* 2021) each reveal complementary aspects of how proteins are organized in cells. Integrating across these multiple approaches could substantially increase proteome coverage and fidelity over what is obtained with any single technique for mapping cell structure, advancing toward the goal of providing a complete view of protein assemblies (Schaffer *et al.* 2025).

In recent years, the field of machine learning has developed a powerful arsenal of approaches for combining multiple types of data collected for a sample (i.e. data modalities) into a general unified representation (i.e. sample embedding) (Radford et al. 2021, Girdhar *et al.* 2023). In the emerging class of approaches known as Foundation models, this representation is learned in a general task-agnostic manner, without being specifically trained for any particular downstream application. Recently, Foundational models have been applied in biology to create integrated embeddings of single-cell sequencing and imaging data (Yang *et al.* 2021, Bao *et al.* 2022) as well as molecular interactions (Forster *et al.* 2022, Nasser and Sharan 2023). To apply these concepts to datasets for mapping subcellular organization, we developed a framework called ProteinProjector, which integrates any number of proteomic datasets to learn a unified protein representation that captures information from each of the original modalities (Fig. 1b).

## 2 Methods

### 2.1 Compilation of HEK293 features

AP-MS interactions generated by the OpenCell project (Cho *et al.* 2022) were downloaded from https://opencell.czbiohub.org/. SEC-MS profiles were generated in a previous study (Heusel *et al.* 2019) and downloaded from the publication site Supplementary Material, available as supplementary data at *Bioinformatics Advances* online. To generate a network, we processed SEC-MS data using the PrInCE software (Stacey *et al.* 2017) and selected protein pairs with precision >0.75. PL-MS interactions generated in the Human Cell Map project (Go *et al.* 2021) were downloaded from humancellmap.org (saint-080922.txt) and filtered for high confidence interactions (BFDR < 0.01). For each of these network-based HEK293 datasets, we used node2vec to generate a 1024-dimensional embedding for each protein (https://github.com/idekerlab/cellmaps_ppi_embedding, *p* = 2, *q* = 1, walk length = 80, number of walks = 10). The images of protein subcellular distribution were also generated by the OpenCell project (Cho *et al.* 2022). An embedding for each image was generated using cytocelf (Kobayashi *et al.* 2022); these 9217-dimensional embeddings were directly downloaded from GitHub (https://github.com/royerlab/cytoself).

### 2.2 ProteinProjector model architecture

ProteinProjector consists of an encoder and a decoder for each modality. The separate embedding vector inputs ($x_{m,i}$ for protein *i* in modality *m*) are compressed by modality-specific encoders ($f_{m}$), yielding 128-dimension vectors $z_{m,i}$:


$$z_{m,i}=\;f_{m}\;(x_{m,i})$$

$$f_{m}\;=L2\mathrm{Norm}(\mathrm{Linear}(\mathrm{ReLU}(\mathrm{Linear}$$

(Dropout(ReLU(Linear(Dropout())))))))

“Dropout” indicates dropout layers (Srivastava *et al.* 2014); “Linear” indicates linear transformation layers; L2Norm indicates L2-normalization; ReLU indicates the Rectified Linear Unit function. The dimensionality of the linear layers is calculated based on the dimensions of the embedding vector inputs. The $z_{m,i}$ embeddings are passed to modality-specific decoders ($g_{m}$), yielding the reconstructed inputs ($y_{m,i}$):


$$y_{m,i}=\;g_{m}(z_{m,i})$$

$$g_{m}=\mathrm{Linear}(\mathrm{ReLU}(\mathrm{Linear}($$

(ReLU(Linear(Dropout())))))

### 2.3 Loss functions

To compute the reconstruction loss *R*, the outputs *y* are compared to the original inputs *x* for each combination of modality pairs. For example, for modalities *a, b:*


$$R_{a,b}=\frac{1}{n}\sum_{i=1}^{n}\;D(x_{a,i},y_{b,i}{)}^{\;}$$

where D denotes the cosine distance (1 - cosine similarity) and *n* is the total number of proteins present in both modalities *a*, *b*. The overall reconstruction loss is then sum of reconstruction losses across each combination of pairs of modalities in the set of all modalities *M*:


$$R=\sum_{a\epsilon M\;}^{\;}\sum_{b\epsilon M}^{\;}R_{a,b}$$

To compute triplet loss *T* for modalities *a, b*, each $z_{a,i}$ is compared to one positive example $z_{b,i}$ (the same protein in the other modality) and one negative example $z_{b,k}$ (a different protein in the other modality; *k* is a randomly sampled protein where *k ≠ i*):


$$T_{a,b}=\frac{1}{N}\sum_{i\epsilon N}^{\;}\max(D(z_{a,i},\;z_{b,i})-\;D(z_{a,i},\;z_{b,k})+\;\varepsilon ,0)$$

where *N* is the set of all proteins shared between *a, b* and ε is the triplet margin. For each batch, the loss $T_{a}$ for modality *a* is computed by randomly selecting a single separate modality *b* (where *a ≠ b*); this selection is performed independently for each batch (see Section 2.4). The overall triplet loss is then:


$$T=\sum_{a\epsilon M}^{\;}T_{a}$$

The full loss function *L* is a weighted sum of the reconstruction and triplet losses:


L=λR+(1-λ)T

### 2.4 Model training

Model parameters were trained with standard neural network learning procedures provided by PyTorch (Paszke *et al.* 2019) v2.0.1, based on backpropagation using the Adam stochastic gradient descent method (Kingma and Ba 2014). Values of hyperparameters were set based on previous work (Schroff *et al.* 2015, Bao *et al.* 2022) without fine-tuning: batch size = 64, λ = 0.5, Adam optimization learning rate = 0.0001, ε = 0.2, dropout = 0.5.

### 2.5 Comparison with original data modalities

**Figure 2.**
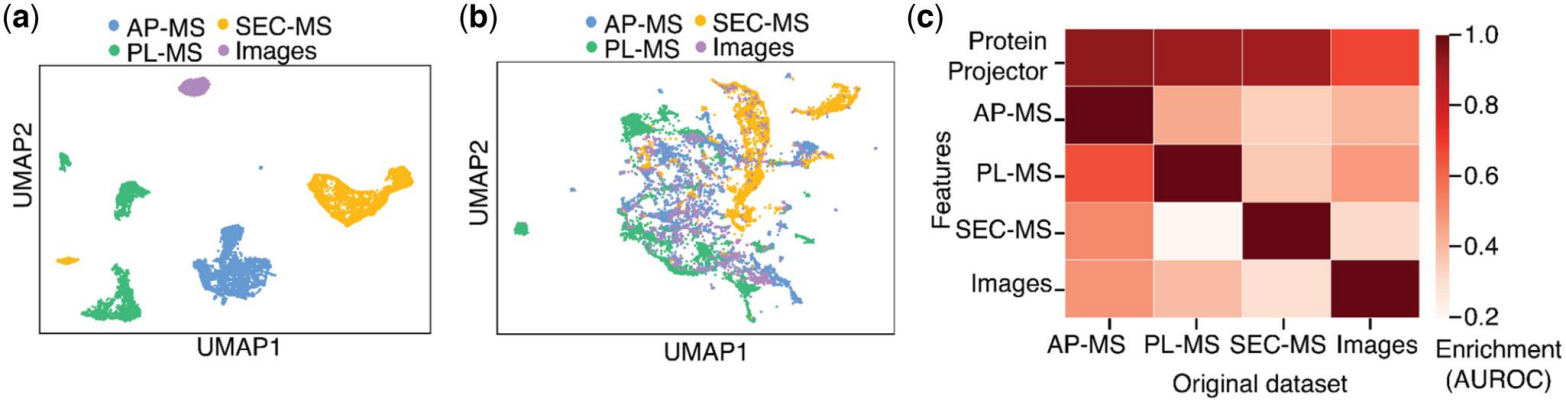
ProteinProjector representations. a) UMAP visualization of ProteinProjector embeddings for each protein prior to training. b) UMAP visualization of ProteinProjector embeddings for each protein after training. c) Agreement of each original data type embeddings (columns) to each other (rows) or to the ProteinProjector embedding (top row). Agreement measured by enrichment of most similar protein pairs in one embedding versus another, defined by AUROC (Section 2).

To compare ProteinProjector embeddings with the original data modalities’ embeddings (Fig. 2c), the primary metric used was the area under the receiver operating characteristic (AUROC), comparing the distribution of ProteinProjector protein proximities for positive vs. negative pairs in each original modality. For each original modality, positive protein pairs were defined as the top 1% of most similar pairs (cosine similarity) in the embeddings (see Section 2.1), and negative pairs were all other pairs.

### 2.6 Protein functional analysis

**Figure 3.**
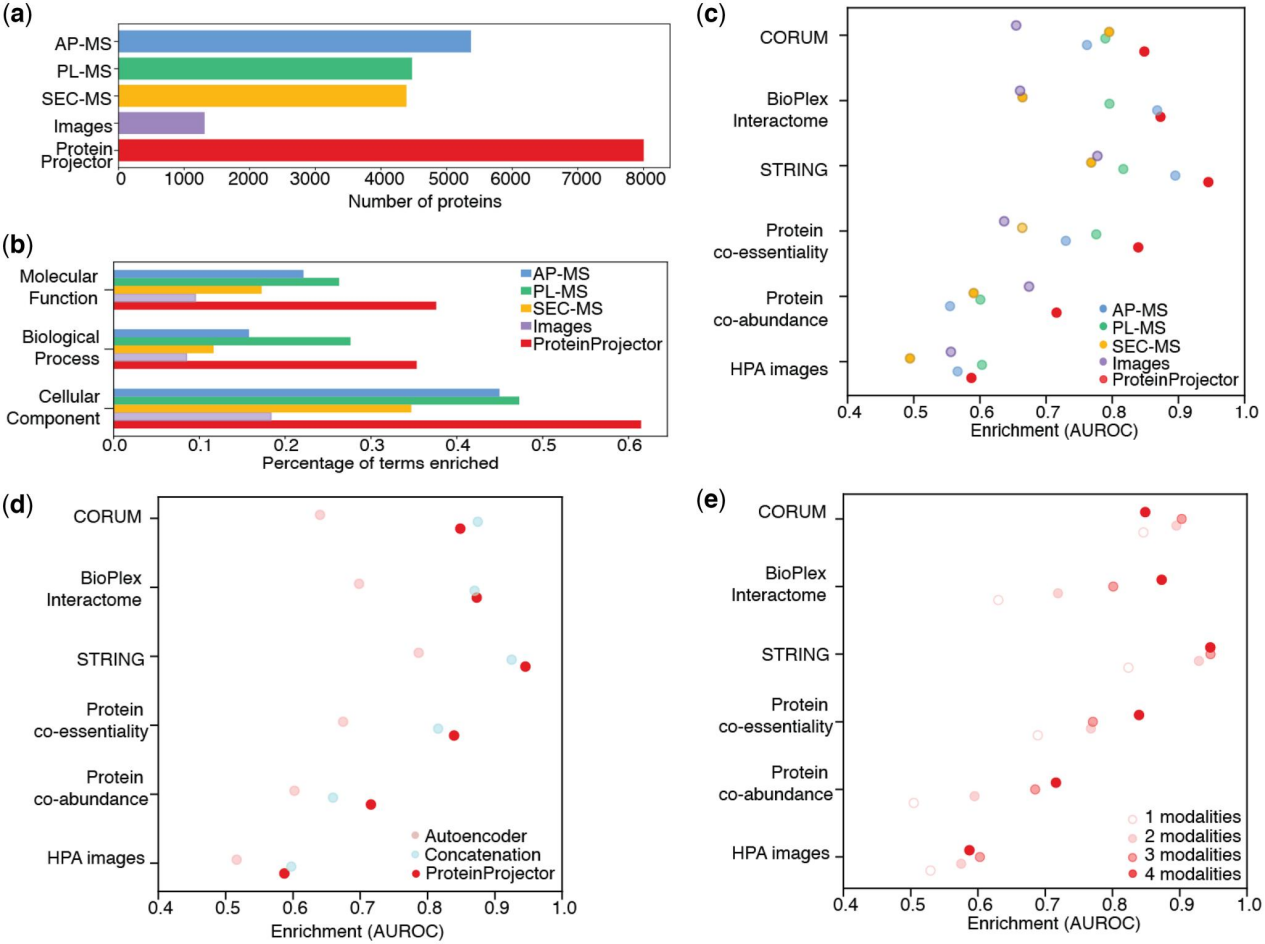
Evaluation of ProteinProjector integration. a) Number of proteins covered in each original modality vs. ProteinProjector. b) Fraction of Gene Ontology terms recovered in similar protein proximities for original modality embeddings and ProteinProjector (Section 2, <1% FDR). c) Degree of enrichment (AUROC, Section 2) of similar protein proximities (colored points, each data modality individually and combined with ProteinProjector) for orthogonal functional and physical association datasets not used in model training (rows), focused on proteins present in all four original datasets. d) Similar to panel (c), comparison of ProteinProjector embeddings to a standard autoencoder integration and to simple concatenation of input features (Section 2). e) Similar to panel (c), but for ProteinProjector embeddings that incorporate increasing numbers of data modalities (colored points).

For each branch of the Gene Ontology (January 2024 release), the number of GO terms covered in ProteinProjector protein proximities was determined as follows. For the set of proteins in each GO term, we determined the distribution of protein proximities for all pairs of these proteins. This similarity distribution was then compared to a null distribution (all pairs of proteins not in any GO term, i.e. assigned to root node only) using a one-sided Wilcoxon rank-sum test with Benjamini-Hochberg correction (Fig. 3a, <1% FDR). For single modalities, the cosine similarities between original embeddings were used (see Section 2.1).

### 2.7 Comparison with orthogonal datasets

CORUM complexes were obtained from NDEx (v4.1, NDEx uuid 764f7471-9b79-11ed-9a1f-005056ae23aa), and pairs of proteins with co-presence in a complex were extracted. BioPlex protein pairs were obtained from NDEx (uuid 6b995fc9-2379-11ea-bb65-0ac135e8bacf). High-confidence STRING v12 pairs were obtained from NDEx (uuid 0b04e9eb-8e60-11ee-8a13-005056ae23aa) For protein co-essentiality pairs, the K562 day-8 perturb-seq dataset was acquired at gwps.wi.mit.edu (BioProject ID PRJNA831566); we computed a pairwise Pearson correlation matrix and extracted the top 1% most similar pairs as interactions. Protein co-abundance data was downloaded from a previous study (Gonçalves *et al.* 2022); we computed a pairwise Pearson correlation matrix and extracted the top 1% most similar pairs as interactions. Other thresholds (top 5%, 10%, and 20%) were evaluated in Fig. 3, available as supplementary data at *Bioinformatics Advances* online. Human Protein Atlas (HPA) data images were downloaded (https://github.com/idekerlab/cellmaps_imagedownloader) and classified using Densenet (Ouyang *et al.* 2019) (https://github.com/idekerlab/cellmaps_image_embedding); we determined pairs of proteins with a shared subcellular compartment. To compare ProteinProjector embeddings with these orthogonal datasets (Fig. 3c–e), the primary metric used was the AUROC for protein pairs positive in the evaluation set (e.g. pairs of proteins in CORUM complex) vs. negative pairs (e.g. pairs of proteins not in CORUM complex). For single modalities, the cosine similarities between original embeddings were used (see Section 2.1). For the concatenation comparison (Fig. 1, available as supplementary data at *Bioinformatics Advances* online), if a protein was missing from a modality, a feature from that modality was randomly selected. For the standard autoencoder comparison, embeddings from each modality were first encoded separately with a linear layer and Rectified Linear Unit (ReLU) activation function to produce a 128-dimensional latent vector. These vectors were then concatenated and passed through an additional linear layer to produce a 128-dimensional latent vector. This latent vector was subsequently decoded back into the respective modalities, and the reconstruction accuracy was evaluated to ensure effective integration. The model was trained with data present in all four modalities for 50 epochs using a batch size of 64 and the Adam optimizer.

### 2.8 Cell map construction

**Figure 4.**
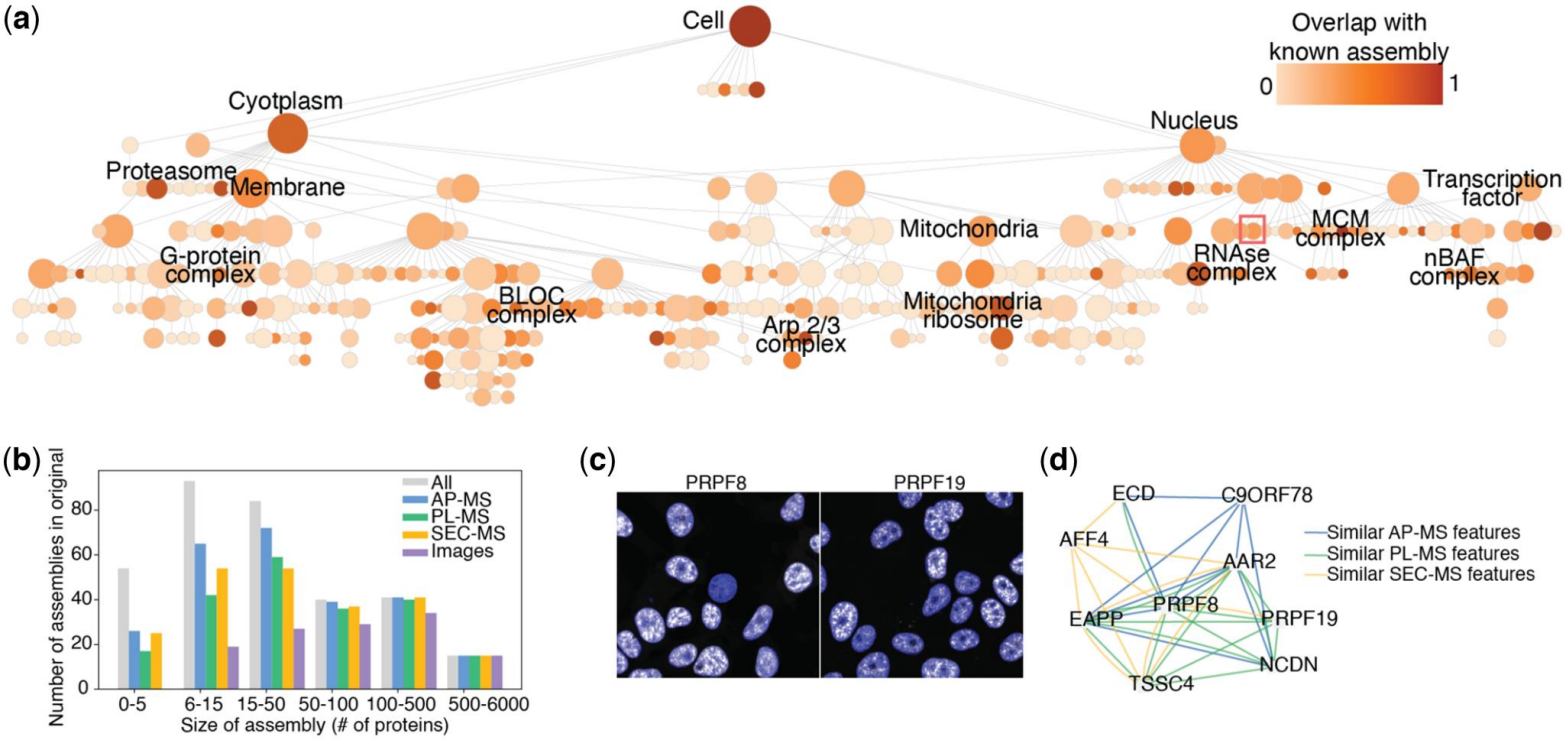
Cell map of protein assemblies in HEK293 based on ProteinProjector. a) Hierarchy of protein assemblies constructed by performing multi-scale community detection on ProteinProjector embeddings. Node shade based on overlap with known subcellular components from GO, CORUM, and HPA (Section 2). Red box denotes assembly highlighted in c, d. b) Number of assemblies with support from original data modalities (Section 2) versus size of assembly in number of proteins. Gray bars denote the total number of assemblies in each size category. c) Live fluorescence cell images for proteins in spliceosome-associated complex present in the imaging dataset (PRPF8 and PRPF19). d) Spliceosome-associated complex supported by similar features (top 1% pairs by cosine similarity) from original data modalities.

We used the Cell Mapping toolkit (https://github.com/idekerlab/cellmaps_generate_hierarchy) to generate a hierarchical cell map of protein assemblies (Fig. 4a, available as supplementary data at *Bioinformatics Advances* online). The ProteinProjector protein proximities were used to generate a series of protein-protein proximity networks in which edges were defined from the most similar 0.2, 0.4, 0.6, 0.8, 1.0, or 5.0% pairs, respectively, yielding six networks total. Pan-resolution community detection was performed in each of these networks using the Hierarchical community Decoding Framework (HiDeF, https://github.com/fanzheng10/HiDeF) (Zheng *et al.* 2021), with a persistence threshold (k) of 10 and a maximum resolution (maxres) of 80, with other parameters kept at default settings. The Cell Mapping toolkit (https://github.com/idekerlab/cellmaps_hierarchyeval) was also used to determine overlap with GO and CORUM terms via a hypergeometric test with Benjamini-Hochberg correction (<1% FDR and Jaccard index >0.2). To determine which assemblies were driven by the original data modalities, we performed the following. For the set of proteins in each assembly we determined the cosine similarity between original embeddings for all pairs of these proteins (see Section 2.1). This similarity distribution was then compared to a null distribution (all pairs of proteins not in any common assembly, i.e. assigned to root node only) using a one-sided Wilcoxon rank-sum test with Benjamini-Hochberg correction (Fig. 4a and b, <1% FDR).

## 3 Results

ProteinProjector employs an encoder–decoder neural network architecture, in which a bank of encoders distills features from each of the separate data modalities collected for a protein into a unified embedding, while a corresponding bank of decoders reconstructs the original features from this shared space. This system is trained in such a way as to minimize error between the reconstructed and original datasets (“reconstruction loss,” Section 2), which encourages accurate data replication. Furthermore, the multiple modalities characterizing a protein are encouraged to occupy embedding coordinates that are similar to one another but distinct from the coordinates of other proteins (“triplet loss,” a type of contrastive learning; Section 2). While ProteinProjector trains from all available data for each protein, a key feature is its tolerance to missing data (i.e. it does not require that a protein is covered by every dataset).

As proof-of-concept, we applied this approach to integrate the growing wealth of protein physical association datasets generated in the human embryonic kidney (HEK)-293 cell line, a common model used for *in vitro* studies of human cell biology. We collected datasets from multiple mass spectrometry techniques including AP-MS (Cho *et al.* 2022), PL-MS (Go *et al.* 2021), SEC-MS (Heusel *et al.* 2019), and protein fluorescent images (Cho *et al.* 2022) (Fig. 1a; Table 1, available as supplementary data at *Bioinformatics Advances* online). These four datasets were supplied to ProteinProjector, which used them to learn the unified embedding space (Section 2). UMAP projection of the embeddings revealed that the modality embeddings are more unified in the latent space after integration with ProteinProjector (Fig. 2a and b). As needed for later applications, we averaged the separate modality ProteinProjector embeddings to produce a single unified embedding per protein. This embedding is then used as a basis for computing protein-protein pairwise cosine similarities, which we call “protein proximities,” for comparison against other datasets.

We first investigated how the ProteinProjector embedding positions protein pairs in comparison to the original data modality embedding (Section 2), prior to training. The ProteinProjector protein proximities showed high levels of enrichment for the most similar pairs in each of the original modalities (Fig. 2c), demonstrating how the ProteinProjector embeddings retain protein proximity information from each original dataset.

We found that the ProteinProjector embedding increases coverage of the proteome, containing more proteins than any single modality alone (Fig. 3a). In particular the ProteinProjector embedding included coordinates for 8004 proteins, versus ∼5500 proteins for AP-MS (the most complete single modality) and ∼1200 proteins for imaging (the least complete modality). As a result of this expanded coverage, we found that ProteinProjector also markedly improves coverage of protein functions. In particular, ProteinProjector embeddings covered the highest fraction of Gene Ontology terms (GO, all three branches) compared to each individual modality (Fig. 3b, Section 2).

We next examined how well the ProteinProjector embedding similarities position protein pairs with high similarity in orthogonal functional and physical datasets not used in training. These datasets included a protein co-essentiality screen, defined as pairs of proteins with similar transcriptional profiles upon CRISPR perturbations (Tsherniak *et al.* 2017); protein co-abundance, defined as pairs of proteins with similar abundances across cell types (Gonçalves *et al.* 2022); interactions in independent AP-MS dataset, the BioPlex interactome in HEK-293 (Huttlin *et al.* 2015, 2021); pairs of proteins with similar Human Protein Atlas immunofluorescence images (HPA) (Thul *et al.* 2017); STRING interactions (Szklarczyk *et al.* 2019); and co-membership in a CORUM complex (Tsitsiridis *et al.* 2023). Compared to individual datasets, the ProteinProjector embedding markedly improved the enrichment (area under the receiver operating characteristic, AUROC, Section 2) between pairs in functional datasets like protein co-abundance and physical datasets such as pairs in the same CORUM complex (Section 2, Fig. 3c). We observed that for the subset of proteins present in all four modalities (580 proteins), concatenation performed close to, or on par with, ProteinProjector (Fig. 3d). However, a core strength of ProteinProjector is that it does not require all modalities present to generate an embedding for each protein. Notably, when considering all proteins measured by at least one modality (8004 proteins, Section 2), we found that ProteinProjector markedly improves upon concatenation in recovery of all external standards (Fig. 1, available as supplementary data at *Bioinformatics Advances* online). This analysis suggested that the ProteinProjector embeddings better capture physical and functional relationships between proteins than any modality alone. Furthermore, ProteinProjector also tended to outperform other strategies for data integration, including simple concatenation of modality features and a standard autoencoder (Fig. 3d). We noticed the observed trends held true at varying thresholds of top 5%, 10%, and 20% of pairs for both the original datasets and when using thresholds for orthogonal datasets (Protein co-essentiality and Protein co-abundance; Figs 2 and 3, available as supplementary data at *Bioinformatics Advances* online).

We analyzed how agreement with the orthogonal datasets varied when comparing ProteinProjector embeddings for proteins covered by all four data modalities versus proteins covered by only one, two or three (Fig. 3e). This analysis revealed that in general, proteins present in greater numbers of modalities show more agreement with the functional and physical datasets. Proteins only present in one or two data modalities still tended to demonstrate a positive enrichment for physical associations such as CORUM, but integrating across modalities tended to improve this effect. To further investigate, we analyzed a set of experiments where ProteinProjector was trained using combinations of two or three modalities. While the ProteinProjector embedding trained with four modalities consistently performs within the top two, other subsets of the four modalities vary in performance depending on the dropped modalities and the external dataset (Fig. 4, available as supplementary data at *Bioinformatics Advances* online).

One downstream application of ProteinProjector is integration of modalities for mapping cell structure in order to robustly identify protein assemblies (Qin *et al.* 2021). We performed multiscale community detection (Zheng *et al.* 2021) on the ProteinProjector protein proximities to construct a global, integrated map of the cell with the union of proteins across all data modalities. This global set of 8,004 proteins was organized into 359 protein assemblies (Fig. 4a), including 147 with high overlap with a known GO cellular component or CORUM term (Section 2). The remaining 212 assemblies were designated as putatively novel assemblies. We assessed each protein assembly for presence of similar original data modality features (Section 2), determining which assemblies were driven by different data modalities (Fig. 4b; Fig. 5, available as supplementary data at *Bioinformatics Advances* online). This analysis revealed 279 assemblies supported by AP-MS evidence, 228 by PL-MS evidence, 244 by SEC-MS evidence, and 125 by image evidence. Of these, 244 assemblies (of which 121 were putatively novel) were informed by more than one modality, providing robust evidence for novel associations. For example, the map revealed a putative nuclear protein assembly involved in RNA splicing with support from multiple original data modalities, including similar images with nuclear localization (Fig. 4c) and interactions across the MS datasets (Fig. 4d), suggesting these protein associations are robustly recovered across experiments.

## 4 Discussion

ProteinProjector presents a flexible framework for integrating multiple proteomics data sources into a low dimensional representation of each protein which can be used for downstream analyses such as mapping subcellular structure. Alternative approaches (e.g. feature concatenation, Fig. 1, available as supplementary data at *Bioinformatics Advances* online) require all modalities to be present for each protein. Additionally, ProteinProjector implements a self-supervised approach which avoids some of the pitfalls of supervised approaches, such as biases toward well-studied proteins.

While an individual proteomics data modality may perform strongly in recovery of a particular external dataset, we find that overall ProteinProjector provides the best performance across a range of external standards (Fig. 3), including the recovery of the largest number of documented subcellular components (Fig. 3b). We note that dropping out modalities has variable effects that depend on the particular modality and external standard used for evaluation. The ability to integrate new data modalities as they become available will enable further investigations of how these modalities excel in characterizing different types of interactions or classes of proteins.

A future avenue for exploration will be to study cases that present conflicts among the different modalities. For example, two proteins may have very similar immunofluorescent images but completely disjoint patterns of protein interactions in AP-MS data, placing tension on the ProteinProjector embedding. Such conflicts raise the question of whether one of the modalities is correct and the other is in error, in which cases such preferences might be learned during model training. Alternatively, inter-modality conflicts could point to different aspects of protein biology, for example stable versus time-dependent properties of the protein or variations in protein localization across cell types.

Deep learning architectures like ProteinProjector could also be useful for translating across data modalities. For example, one might wish to use a relatively rapid proteome profiling with SEC-MS to predict the protein interactions that would be expected to result from lower throughput techniques such as AP-MS. Future studies may further explore the ProteinProjector modeling framework to determine which aspects of the multimodal architecture are critical to its performance, to compare different architectures, and to assess which models apply best to specific biological datasets or problems. ProteinProjector can also be readily extended to add even greater numbers of data modalities or protein features, including direct incorporation of protein sequence or structure.

## Supplementary Material

vbaf266_Supplementary_Data

## Data Availability

AP-MS interactions generated by the OpenCell project (Cho *et al*., 2022) were downloaded from https://opencell.czbiohub.org/. SEC-MS profiles were downloaded from the publication site Supplementary Material in Heusel *et al*. (2019). PL-MS interactions generated in the Human Cell Map project were downloaded from humancellmap.org (saint-080922.txt). OpenCell image embeddings were directly downloaded from https://github.com/royerlab/cytoself. CORUM complexes were obtained from NDEx (v4.1, NDEx uuid 764f7471-9b79-11ed-9a1f-005056ae23aa). BioPlex protein pairs were obtained from NDEx (uuid 6b995fc9-2379-11ea-bb65-0ac135e8bacf). High-confidence STRING v12 pairs were obtained from NDEx (uuid 0b04e9eb-8e60-11ee-8a13-005056ae23aa). For protein co-essentiality pairs, the K562 day-8 perturb-seq dataset was acquired at gwps.wi.mit.edu (BioProject ID PRJNA831566). Protein co-abundance data was downloaded from publication site Supplementary Material in Gonçalves *et al*. (2022). Human Protein Atlas (HPA) images were downloaded from proteinatlas.org.
