# Supplementary material for: Unifying proteomic technologies with ProteinProjector
Source: Bioinform Adv. 2025 Oct 22;5(1):vbaf266. doi: 10.1093/bioadv/vbaf266 (PMC12680973; doi:10.1093/bioadv/vbaf266)
Supplement: vbaf266_Supplementary_Data [file vbaf266_supplementary_data.pdf]

Supplementary Figures

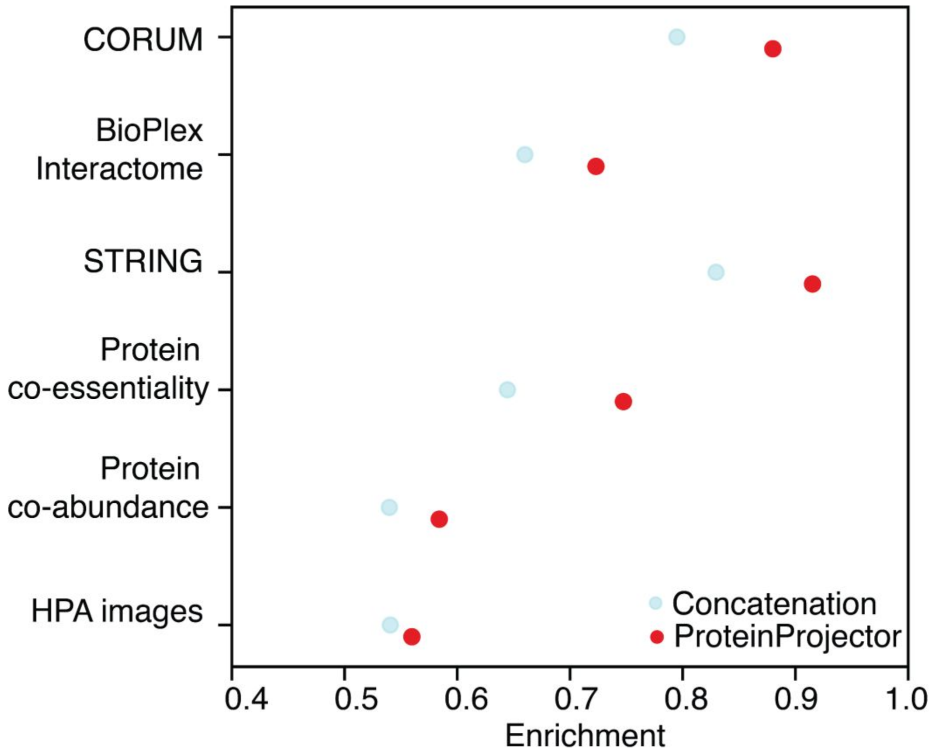

Supplementary Fig. 1. Comparison of ProteinProjector embeddings to simple concatenation of input features using the union of all proteins present in the four modalities (Methods).

Only

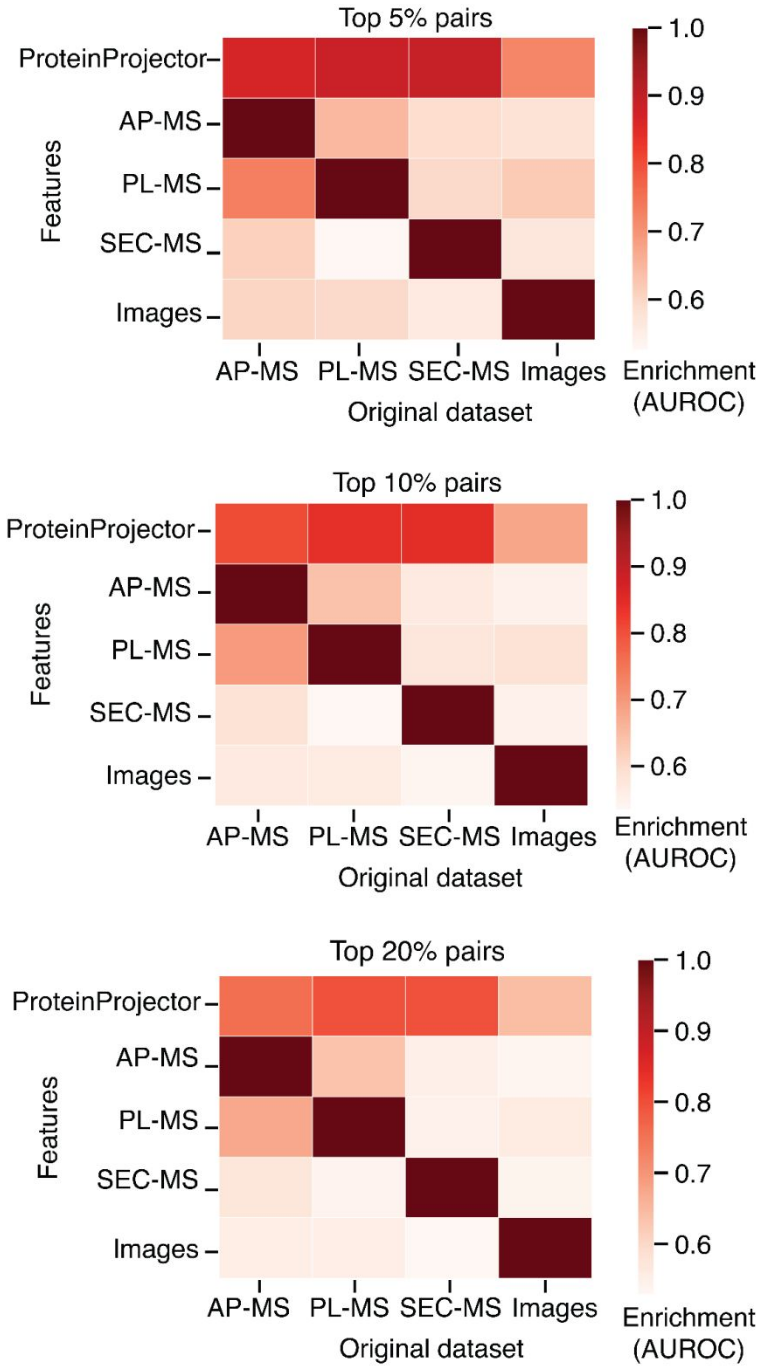

Supplementary Fig. 2 Agreement (white-to-red color gradient) of each original data type embeddings (columns) to each other (rows) or to the ProteinProjector embedding (top row). Agreement measured by enrichment of most similar protein pairs at different thresholds (5%, 10%, and 20%) in one embedding versus another, defined by AUROC (Methods).

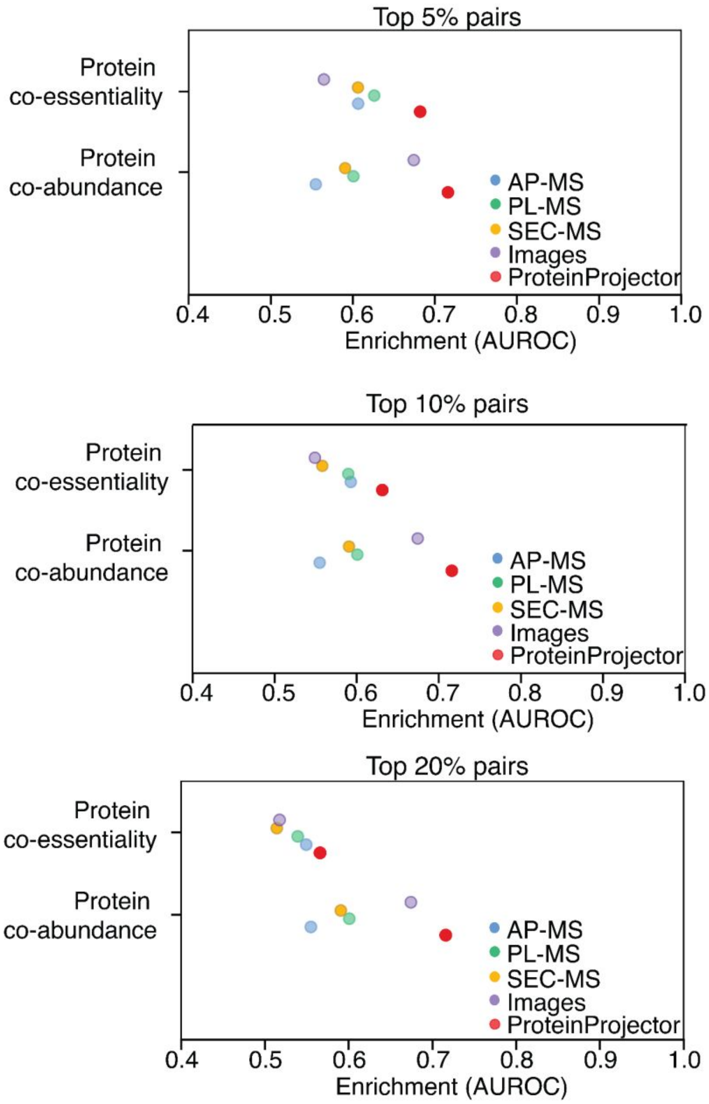

Supplementary Fig. 3. Degree of enrichment (AUROC, Methods) of similar protein proximities (colored points, each data modality individually and combined with ProteinProjector) for orthogonal functional and physical association datasets not used in model training (rows), focused on proteins present in all four original datasets. Different thresholds were used for orthogonal datasets Protein co-essentiality and Protein co-abundance to define protein pairs (top 5%, 10%, and 20% most similar pairs, Methods).

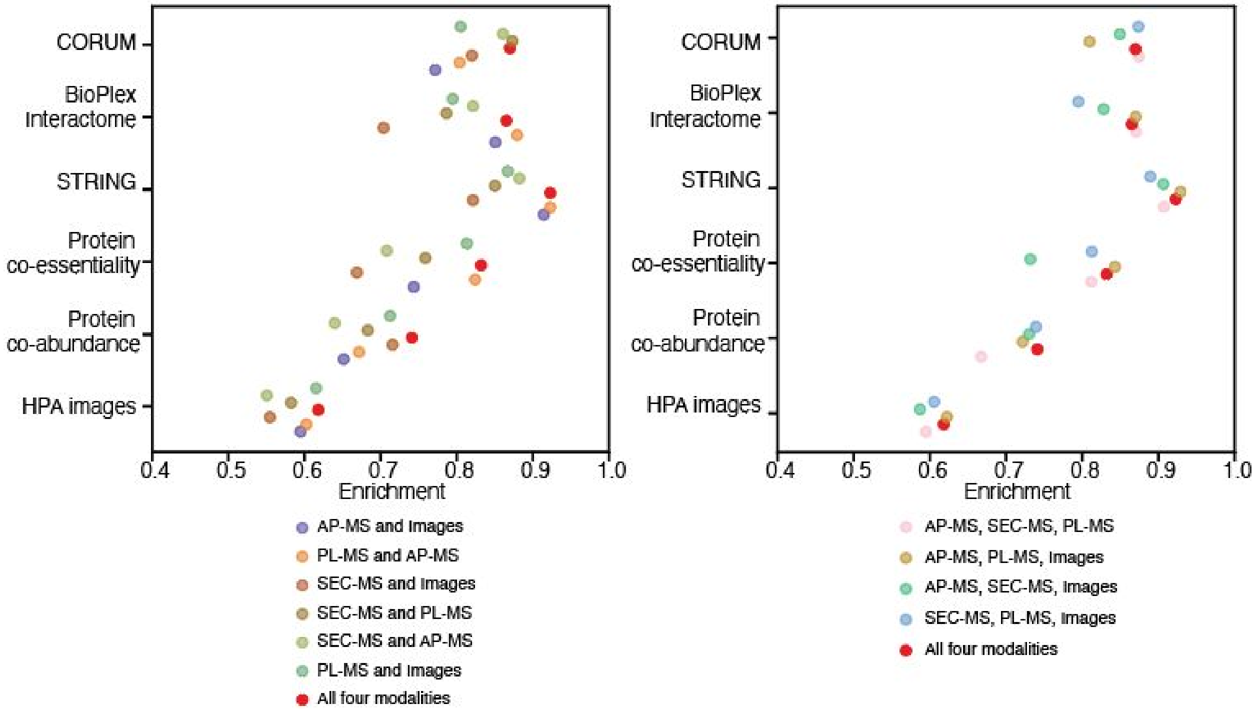

Supplementary Fig. 4. Degree of enrichment (AUROC, Methods) of similar protein proximities (colored points, each data modality individually and combined with ProteinProjector) for orthogonal functional and physical association datasets not used in model training (rows), using different subsets of the modalities in training ProteinProjector.

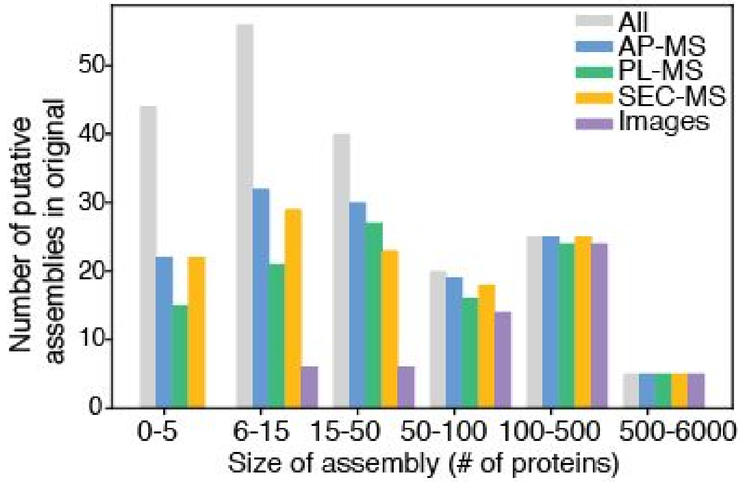

Supplementary Fig 5. Number of putative assemblies with support from original data modalities (Methods) versus size of assembly in number of proteins. Gray bars denote the total number of assemblies in each size category

Supplementary Tables

Modality	Total	CORUM	BioPlex Interactome	STRING	Protein co-essentiality	Protein co-abundance	HPA images
**AP-MS**	5372	2351	4759	2862	783	1983	4266
**PL-MS**	4473	1838	4133	2163	607	1798	3775
**SEC-MS**	4387	1976	4199	2370	700	1961	3604
**Images**	1311	728	1252	850	226	587	1109

Supplementary Table 1. Number of proteins in each modality and overlap with each orthogonal dataset analyzed.

For Review Only
